# Assessment of Knowledge towards Radiation Protection Measures among Newly Graduated Dentists from Egypt and the Kingdom of Saudi Arabia: A Questionnaire-Based Cross-Sectional Study

**DOI:** 10.3390/dj10060095

**Published:** 2022-06-01

**Authors:** Soha Mohamed Ali Basha, Munerah Saleh BinShabaib, Shatha Subhi ALHarthi

**Affiliations:** 1 Department of Basic Dental Sciences, College of Dentistry, Princess Nourah Bint Abdulrahman University, Riyadh 11671, Saudi Arabia; smbasha@pnu.edu.sa; 2 Department of Preventive Dental Sciences, College of Dentistry, Princess Nourah Bint Abdulrahman University, Riyadh 11671, Saudi Arabia; msbinshabaib@pnu.edu.sa

**Keywords:** ALARA, radiation hazards, ionizing radiation, newly graduated dentists

## Abstract

**Background**: It is hypothesized that knowledge towards ionizing radiation (IR) protection measures is lacking among newly graduated dentists from Egypt and Saudi Arabia. The purpose of the present questionnaire-based study was to compare the IR protection knowledge among newly graduated dentists from Egypt and the Kingdom of Saudi Arabia (KSA). Methods: The present investigation was a questionnaire-based study and had a cross-sectional observational analytical design. Newly graduated dentists were defined as individuals who had graduated from a credentialed dental institution within the past 36-months. The questionnaire was related to knowledge about radiation safety, awareness and practices. The questionnaire comprised of 17 multiple choice questions. Questionnaires in which all the 17 questions were not answered or had missing pages were excluded. Odds ratios were computed for the number of correct and incorrect responses and 95% confidence intervals were determined. Individuals that provided up to 40%, 41% to 70% and >70% correct answers were categorized as having “low competence”; “moderate competence” and “high competence” in IR knowledge. *p*-values less than 0.05 were considered statistically significant. Results: The mean percentage of correct answers provided by dentists from Egypt and KSA were 56.9% and 67.4%, respectively. There was no statistically significant difference in the percentages of correct responses for the 17 questions submitted by respondents from Egypt and KSA. The overall percentage of correct responses ranged between 40.2–71.2% and 45.4–81.4% for respondents from Egypt and KSA, respectively. The odds ratios and 95% confidence intervals showed no statistically significant correlation in the responses from respondents from Egypt and KSA for each of the 17 questions addressed. Conclusion: Newly graduated dentists from Egypt and KSA are moderately competent regarding IR doses and related safety measures. It is recommended that modifications in the undergraduate dental education curriculum with emphasis on IR safety and practice would help enhance the knowledge and competence of students and newly graduated dentists. Moreover, routine continuing dental education seminars/programs may help enhance the IR knowledge of dental students and dentists.

## 1. Introduction

The clinical evaluation of patients presenting with oral symptoms and/or diseases is usually the first step towards diagnosis and treatment planning. However, visual assessment of dentoalveolar and craniofacial tissues bears limitations. For instance, although dental caries is often visually identified during clinical examinations; the possible involvement of pulpal and peri-radicular tissues is challenging to determine through clinical examinations [1,2,3,4]. Likewise, interproximal caries is more often diagnosed on bitewing radiographs than clinically [1]. Moreover, in the field of implant dentistry, correlation of the alveolar bone with vital anatomical structures such as the maxillary sinus and inferior alveolar nerve warrants an in-depth radiographic evaluation using panoramic radiographs and cone-beam computed tomographic scans [4]. Furthermore, radiographic evaluation is essential for the diagnosis of periodontal and peri-implant osseous morphology and related diseases [5,6]. In this context, the ability of ionizing radiation (IR) to penetrate tissues and display images that are not visible to the human eye is plausible. However, safety and precautionary measures must be taken into consideration during IR as it may latently jeopardize living tissues [7]. It is important for professionals to be aware of the criteria associated with accurate imaging. However, there are a number of safety and precautionary measures that need to be taken into consideration while taking dental and craniofacial radiographs. It has been reported that IR, through the elaboration of free radicals, causes changes in the DNA strands and ensues damage to living tissues [8,9]. In addition, exposure to IR in the absence of safety measures has been linked with infertility among males [9].

In a questionnaire-based study from Poland, Furmaniak et al. [10] assessed IR protection awareness among undergraduate dental students (UDS) from a medical university in Warsaw. Results from 301 returned questionnaires showed that UDS had limited knowledge of IR protection [10]. However, results from another questionnaire-based study from Nepal showed otherwise [11]. In a study by Garg and Kapoor [11], 100 questionnaires related to knowledge on IR protection were distributed to fourth year dental students. The results showed that 85% of the respondents were knowledgeable about the hazards associated with IR and the safety measures that are needed to minimize related health hazards [11]. According to An et al. [12], dentists that have short experience since graduation are less knowledgeable towards IR protection measures compared with experienced dentists. Since dentists are at risk of IR exposure and related hazards throughout their career, it is imperative for dentists to be critically aware of the IR exposure parameters and protective measures in order to minimize the undesirable effects of IR.

Based upon evidence from indexed databases, there is a dearth of studies from Middle Eastern countries that have investigated the knowledge of dentists regarding IR protection. In the present study, it is hypothesized that newly graduated dentists from Egypt and the Kingdom of Saudi Arabia (KSA) are comparably knowledgeable regarding IR protection. Therefore, the aim of the present questionnaire-based study was to compare the IR protection knowledge among newly graduated dentists from Egypt and KSA.

## 2. Materials and Methods

### 2.1. Ethics Statement

Ethical approval was obtained from the research committee of Pharos University in Alexandria, Egypt; and the Institutional Review Board (IRB) of Princess Nourah Bint Abdulrahman University, KSA (H-01-R-059). To keep the responses completely anonymous, participants were not requested to sign or initial a consent form. Participation was completely voluntary and withdrawal was not associated with any form of penalization or consequences.

### 2.2. Study Design

The present investigation was a questionnaire-based study and had a cross-sectional observational analytical design.

### 2.3. Participants and Study Groups

Newly graduated dentists were included in the present study. Newly graduated dentists were defined as individuals who had graduated from a credentialed dental institution within the past 36-months [13]. Respondents in Group-1 were recruited from a dental college in Cairo, Egypt (Group-1); and individuals in Group-2 comprised of respondents from a dental college in Riyadh, KSA (Group-2). An invitation letter that explained the purpose of the present study was dispatched via postal mail to female respondents from both institutions. Personal information relating to name, residential address, personal contact number, email addresses etc., were not requested. Participants willing to participate were requested to return the questionnaire in a return stamped envelope, which was sent to all individuals. Returning the completed questionnaire was considered consent for participation.

### 2.4. Information Sheet and Questionnaire

An information sheet, printed in Arabic and English, which explained the purpose/objectives of the present study was sent to all individuals along with the questionnaire. Moreover, the information sheet also stated that the outcomes and results of this survey would not have any impact on the dental licensure of the respondent and that no personal information which could have identified the respondent was being collected. The information sheet clearly mentioned that participation is completely voluntary and individuals not willing to participate should return the sent documents without entering any information. The information sheet provided with the questionnaire also stated that the respondents should use their existing knowledge to respond to the questions without consulting literature and/or peers.

Validation and feasibility of the questionnaire was performed via distribution to 20 randomly selected respondents, (10 respondents from Egypt and 10 respondents from KSA, respectively). The questionnaire was related to knowledge about radiation safety, awareness and practices. The questionnaire comprised of 17 multiple choice questions (Table 1). Printed versions of the questionnaire and a prepaid return envelope with a return address were dispatched in a sealed package by postal mail to 300 newly graduated dentists from Egypt and 300 newly graduated dentists from KSA, respectively. Individuals that provided up to 40%, 41% to 70% and >70% correct answers were categorized as having “low competence”; “moderate comtetence” and “high competence” in IR knowledge. This scale was developed on the basis of a study by Koole et al. [14] in which the competence levels of undergraduate dental students were assessed.

### 2.5. Exclusion Criteria

Questionnaires in which all 17 questions were not answered or had missing pages were excluded from the present investigation.

### 2.6. Statistical Analysis

Data were entered into Microsoft Excel sheets by an investigator who was blinded to the study groups. Statistical analysis was performed using a software program (IBM SPSS software package version 20.0. Chicago, IL, USA) by a blinded Statistician. Patient’s age and duration since graduation were presented as means ± standard deviations. The percentages of correct responses between respondents from Egypt and KSA were compared using the student *t*-test. Odds ratios were computed for the number of correct and incorrect responses and 95% confidence intervals were determined. *p*-values less than 0.05 were considered statistically significant.

## 3. Results

### 3.1. Questionnaire

Of the questionnaires dispatched to newly graduated dentists from Egypt (*n* = 300) and KSA (*n* = 300), 288 (96%) and 275 (91.7%), respectively were returned. None of the returned questionnaires were discarded due to missing information. The mean ages of the participants from Egypt and KSA were 30.2 ± 2.3 and 31.04 ± 1.4 years, respectively. All respondents were females. The mean duration (in months) since graduation among respondents from Egypt and KSA were 24.6 ± 2.2 and 25.4 ± 3.05 months, correspondingly (Table 2).

### 3.2. Responses to Questionnaire

**Figure 1:**
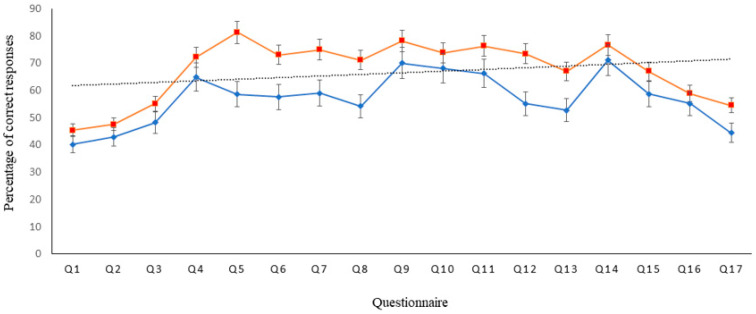
The percentages of correct responses for the 17 questions submitted by respondents from Egypt (red) and the Kingdom of Saudi Arabia (blue).

The percentages of correct responses for the 17 questions submitted by respondents from Egypt and KSA are shown in Figure 1. There was no statistically significant difference in the percentages of correct responses for the 17 questions submitted by respondents from Egypt and KSA. The overall percentage of correct responses ranged between 40.2–71.2% and 45.4–81.4% for respondents from Egypt and KSA, respectively. The mean percentage of correct answers provided by dentists from Egypt and KSA were 56.9% and 67.4%, respectively; and hence were categorized as having moderate competence in IR knowledge.

### 3.3. Correlation between Correct Responses among Respondents from Egypt and KSA

The odds ratios and 95% confidence intervals showed no statistically significant correlation in the responses from respondents from Egypt and KSA for each of the 17 questions addressed (Table 3).

## 4. Discussion

Awareness and implementation of protection protocols in relation to exposure to IR is essential for patient and provider safety [15,16,17]. The present questionnaire-based study was based on the hypothesis that newly graduated dentists from dental institutions in Egypt and KSA are comparably knowledgeable regarding IR protection. The results are in accordance with this hypothesis as there was no significant difference in the numbers of correct responses given by respondents from both institutions. However, an alarming finding of the present investigation is that there was a notable variation in the numbers of correct answers provided for each question. In other words, none of the questions were accurately answered by all respondents from either of the institutions. Moreover, there was no statistically significant difference in the correct responses registered by dentists that have graduated from a dental university in Egypt or KSA (Table 3). Here, it is pertinent to mention that by no means do the authors intend to discriminate or challenge the respondents on their existing knowledge of oral radiology and related safety protocols. However, this outcome does raise concerns regarding the quality and extent of academic knowledge provided to students prior to graduation. This concern is not only limited to dentists graduating from institutions in the Middle East, but also from other countries.

The authors emphasize the results from a questionnaire-based study from Poland in which Furmaniak et al. [10] investigated IR awareness among graduated dentists and undergraduate students from a Medical University in Warsaw. The questionnaire used in this study [10] comprised of 13 multiple choice questions that were administered to 200 dentists and 100 dentistry students. The results showed that the response rate was low as nearly 50% of the questionnaires were not returned for assessment. In this study [10], the mean percentage of correct answers provided by dentists was 64% and the authors concluded that knowledge of dentists and dental studies students towards oral radiology parameters and related protective measures is inadequate [10]. The authors applaud the results reported by Furmaniak et al. [10] Although in the present investigation the total number of respondents (from Egypt and KSA) was high (*n* = 563 respondents) and the response rate was over 90% from respondents from both countries, the mean percentages of correct answers provided by respondents from Egypt (~57%) and KSA (~67%) were comparable to those reported by Furmaniak et al. [10]. This suggests that there is a dire need to implement an educational platform to enhance the knowledge of dentists and graduating students towards IR parameters and protective protocols. In a survey conducted in Ireland, Soye and Paterson [18] suggested that adequate training increases awareness about IR roses and related protective measures during radiological procedures. Similarly, Gallagher et al. [15] recommended that there is a dire need to ensure that academic as well as practical training programs should be provided to oral healthcare providers working with IR. The author further proposed that continuing education courses and routine training after graduation can help to keep providers up to date with current developments and protective strategies implemented in the discipline of radiological sciences. The implementation of such regimens may not only help achieve accurate diagnosis to facilitate treatment but may also ensure the safety of patients and operators. The authors of the present study also evaluated the questions for which the least number of correct answeres were reported. After a careful re-evaluation of the responses, it was observed that Q1 (What does ALARA stand for?), Q7 (What is personal Dosimeter?), Q12 (While taking intraoral radiograph, what is the ideal distance between the operator and the X-ray tube?) and Q16 (What is the occupational radiation dose limit for the X-ray workers?) had the lowest number of correct answers provided by respondents from both institutions. This suggests that further efforts should be made to educate undergraduate dental students regarding technical radiological parameters including IR safety and selecting patients for *radiographic* examinations, and IR exposure parameters. Under- and post-graduate dental continuing education programs and seminars may play a role in enhancing the IR knowledge of students and dentists.

A major concern in the present investigation was to minimize factors that could have led to respondent identification that may have possibly biased the reported results. This step was taken on the perception that in the case that a respondents’ identity was disclosed via the questionnaire this could have either compelled them from either discarding the invitation or possibly consulting literature and/or peers before responding to the questionnaire. We could have sent all questionnaires by email but upon return the identity of the resident could have been disclosed. In order to keep the responses completely anonymous, the authors decided to send the questionnaires via post with prepaid return envelops. Moreover, the statistical analysis of the datasheets was performed by an investigator who was blinded to the study groups. Based upon such measures, the authors perceive that the likelihood of the reported results being biased is minimal.

All respondents were females in the current study. This may be considered a limitation of the present study; however, it is worth mentioning that all newly graduated dentists participating from KSA were graduates of the Princess Nourah Bint Abdulrahman University, which is a public women’s university where all faculty/administrative members are females. In order to minimize the risk of gender bias, newly graduated female respondents were recruited from the Pharos University in Alexandria, Egypt. Further studies involving both male and female newly graduated dentists from KSA and Egypt are needed to identify the IR knowledge of respondents from the Middle-Eastern region.

## 5. Conclusions

Newly graduated dentists from Egypt and KSA are moderately competent regarding IR doses and related safety measures. It is recommended that modifications in the undergraduate dental education curriculum with emphasis on IR safety and practice would help enhance the knowledge and competence of students and newly graduated dentists. Moreover, routine continuing dental education seminars/programs may help enhance the IR knowledge of dental students and dentists.

## Figures and Tables

**Table 1:** Questionnaire.

Serial #	Questions
Q1	What does ALARA stand for?
Q2	Do all human tissues have the same radio-sensitivity?
Q3	Does a routine radiographic examination with a six-month interval prevent the stochastic effect of radiation? (Yes/No)
Q4	Does digital radiography require less radiographic exposure than the conventional type? (Yes/No)
Q5	Does Magnetic Resonance Imaging (MRI) emit ionizing radiation? (Yes/No)
Q6	To achieve maximal patient protection, what is the ideal shape of the collimator?
Q7	What is personal Dosimeter?
Q8	During radiographic exposure, how is the dental X-ray tube focused?
Q9	During intraoral radiology, which part of the patient’s body should be protected?
Q10	How often should lead aprons be used?
Q11	In which trimester are radiographs contraindicated?
Q12	While taking an intraoral radiograph, what is the ideal distance between the operator and the X-ray tube?
Q13	Which type of film requires less radiation exposure?
Q14	What is the ideal position for the dentist to stand in while taking an intraoral radiograph?
Q15	What does CBCT stand for?
Q16	What is the occupational radiation dose limit for the X-ray workers?
Q17	Identify the symbol for radiation (four symbols shown).

**Table 2:** Demographic details of the study cohort.

Parameters	Newly Graduated Dentists from Egypt	Newly Graduated Dentists from the Kingdom of Saudi Arabia
Number of questionnaires sent	300 questionnaires	300 questionnaires
Number of questionnaires returned (*n*) (%)	288 (95%)	275 (91.7%)
Gender of respondents (Female)	288	275
Mean age of respondents in years	30.2 ± 2.3 years	31.04 ± 1.4 years
Duration since graduation	24.6 ± 2.2 months	25.4 ± 3.05 months

**Table 3:** Odds ratios and 95% confidence intervals for correct and incorrect responses to the questionnaire.

Serial #	Question	Correct Response	Incorrect Response	Odds Ratio (95% Confidence Interval)	*p*-Value
Q1	Respondents from Egypt	116	172	0.809 (0.52–0.88)	0.172
	Respondents from KSA	125	150		
Q2	Respondents from Egypt	124	164	0.832 (0.57–0.88)	0.125
	Respondents from KSA	131	144		
Q3	Respondents from Egypt	139	149	0.755 (0.47–0.82)	0.162
	Respondents from KSA	152	123		
Q4	Respondents from Egypt	187	101	0.815 (0.56–0.89)	0.182
	Respondents from KSA	200	88		
Q5	Respondents from Egypt	169	119	0.323 (0.25–0.64)	0.273
	Respondents from KSA	224	51		
Q6	Respondents from Egypt	166	122	0.5 (0.28–0.62)	0.281
	Respondents from KSA	201	74		
Q7	Respondents from Egypt	170	118	0.48 (0.29–0.58)	0.162
	Respondents from KSA	206	69		
Q8	Respondents from Egypt	156	132	0.48 (0.35–0.67)	0.25
	Respondents from KSA	196	79		
Q9	Respondents from Egypt	202	86	0.64 (0.48–0.71)	0.12
	Respondents from KSA	215	59		
Q10	Respondents from Egypt	196	92	0.76 (0.48–0.82)	0.18
	Respondents from KSA	203	72		
Q11	Respondents from Egypt	191	97	0.6 (0.38–0.88)	0.15
	Respondents from KSA	210	65		
Q12	Respondents from Egypt	159	129	0.44 (0.24–0.58)	0.23
	Respondents from KSA	202	73		
Q13	Respondents from Egypt	152	136	0.55 (0.41–0.69)	0.22
	Respondents from KSA	184	91		
Q14	Respondents from Egypt	205	83	0.74 (0.58–0.82)	0.09
	Respondents from KSA	211	64		
Q15	Respondents from Egypt	169	119	0.7 (0.52–0.83)	0.08
	Respondents from KSA	184	91		
Q16	Respondents from Egypt	159	129	0.86 (0.64–0.95)	0.11
	Respondents from KSA	162	113		
Q17	Respondents from Egypt	128	160	0.66 (0.51–0.72)	0.09
	Respondents from KSA	150	125		

KSA: the Kingdom of Saudi Arabia.

## Data Availability

Data is available at reasonable request.
